# Multimodal Therapy for Locally Advanced Scrotal Cancer: A Case Report With a Literature Review

**DOI:** 10.7759/cureus.62544

**Published:** 2024-06-17

**Authors:** Meriem Bouabid, Berhili Soufiane, Ahmed Bensghier, Mohamed Moukhlissi, Loubna Mezouar

**Affiliations:** 1 Radiation Oncology, Centre Hospitalier Universitaire Mohammed VI, Oujda, MAR; 2 Radiotherapy, Centre Hospitalier Universitaire Mohammed VI, Oujda, MAR

**Keywords:** prognostic, radiotherapy, chemotherapy, surgery, scrotal cancer

## Abstract

Cancer of the scrotum is a rare tumor. It is often not diagnosed early, and its treatment poses difficulties for practicing oncologists. We report the case of a patient treated for locally advanced squamous cell carcinoma (SCC) of the scrotum, with a review of the literature. It's about a 66-year-old man, who had been complaining two years ago of an ulcerated lesion at the scrotal region neglected by the patient. However, he presented with a large ulceration on the left inguinal region, for which, the pathological study showed an SCC. CT scan found a left inguinal tumoral process invading the adjacent soft tissues and infiltrating the left spermatic cord without distant localizations. Surgical excision of the inguinal lesion was considered unfeasible, then, an excision of the scrotal lesion was done and the pathological study identified an SCC of the scrotum with clear margins classified T1 N3 M0. Given that inguinal lymph node dissection (LND) was not feasible, concomitant chemo-radiotherapy was indicated at a total dose of 70 Gy in 35 fractions of 2 Gy concomitantly with weekly platinum-based chemotherapy, with good evolution after four months.

## Introduction

Scrotal cancer is a rare disease with an incidence accounting for 1% of malignant tumors. Its treatment poses difficulties for practicing oncologists. The main risk factor is the human papillomavirus (HPV) infection. There are other factors including chronic scrotal inflammation, ultraviolet radiation, smoking, and low socioeconomic status [1].

We report the case of a patient treated at the Hassan II Regional Oncology Center of Oujda for loco-regionally advanced cancer of the scrotum, with a review of the literature to assess the epidemiological, clinical, therapeutic, and prognostic features of this pathology.

## Case presentation

**Figure 1:**
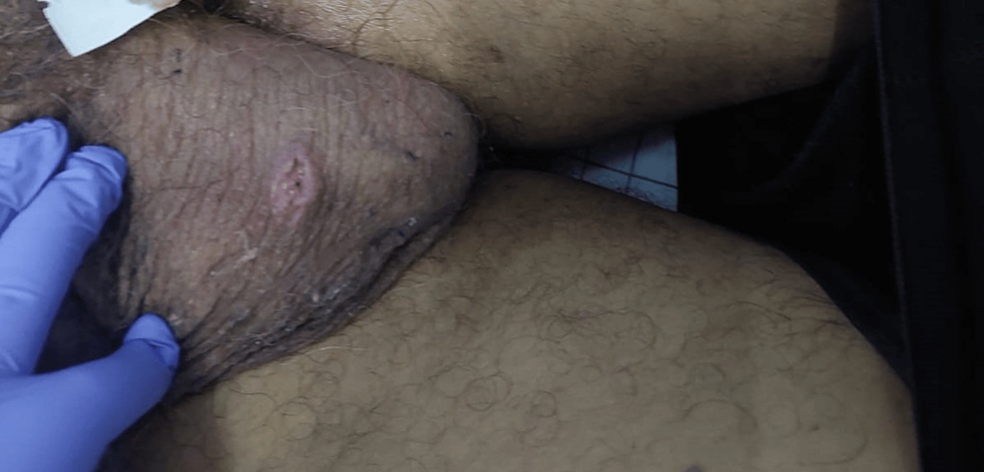
An ulcerated scrotal lesion that had been neglected by the patient.

**Figure 2:**
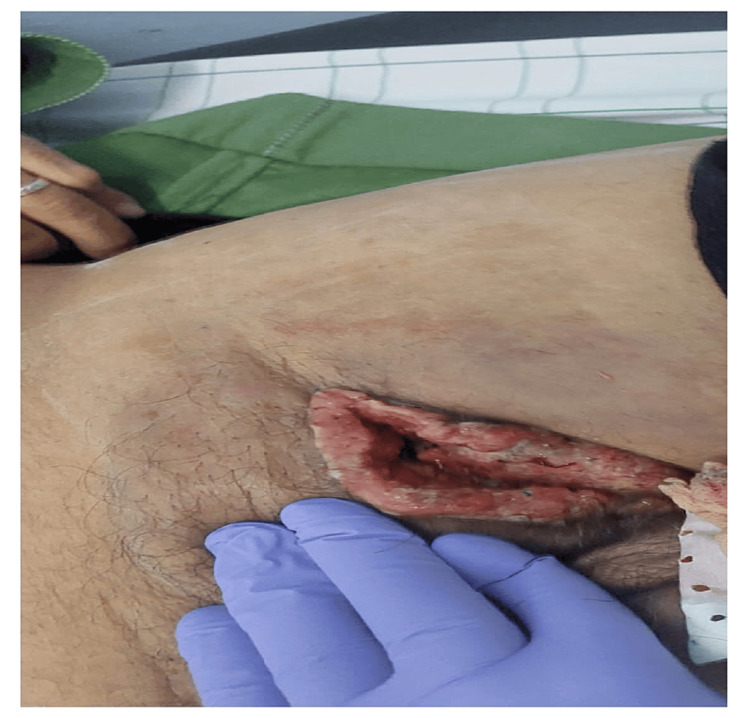
A mass in the left inguinal region, progressively increasing in size and becoming ulcerative.

The patient was 66 years old, a chronic smoker whose family history included three sisters treated for breast cancer and a brother treated for prostate cancer. Two years before the patient's admission to the center, he presented with an ulcerated scrotal lesion that had been neglected by the patient (Figure 1). Currently, he presented with a mass in the left inguinal region, progressively increasing in size and becoming ulcerative and painful (Figure 2), which prompted the patient to seek medical advice. The patient wasn't taking any therapy (weight: 80 kg; height: 1.80 m).

**Figure 3:**
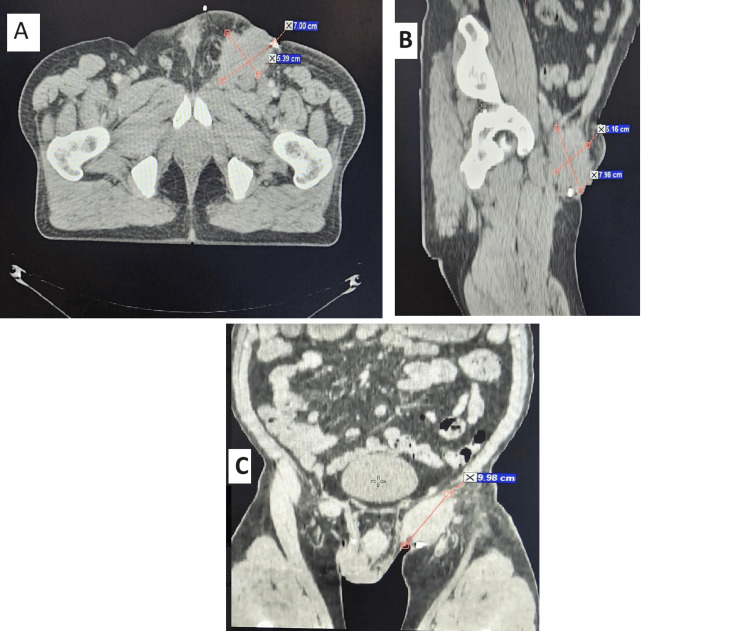
Axial (A), sagittal (B), and frontal (C) CT scan identified a left inguinal tumoral process invading the adjacent soft tissues and infiltrating the left spermatic cord with right inguinal adenopathy and left external iliac adenopathy.

A computed tomography (CT) scan (Figure 3) identified a large left inguinal tumoral process invading the adjacent soft tissues and infiltrating the left spermatic cord with right inguinal adenopathy and left external iliac adenopathy without distant localization. A scrotal ultrasound was also done which found no deep abnormalities in the testicles adjacent to the scrotal ulceration.

A biopsy of the inguinal mass was done and the anatomopathological study was in favor of a well-differentiated, keratinizing, infiltrating squamous cell carcinoma (SCC).

Subsequently, surgical excision of the scrotal lesion was done; and the anatomopathological study identified an SCC of the scrotum with the same characteristics as the inguinal specimen, 2 cm in size, with no vascular embolism (EV-), no perineural invasion (EPN-), and non-tumor margins (R0).

The disease was classified as T2N+M0 stage 3, according to the Lowe modification of the system proposed by Ray and Whitmore. The distant metastasis was evaluated with a chest abdomen and pelvis CT scan.

**Figure 4:**
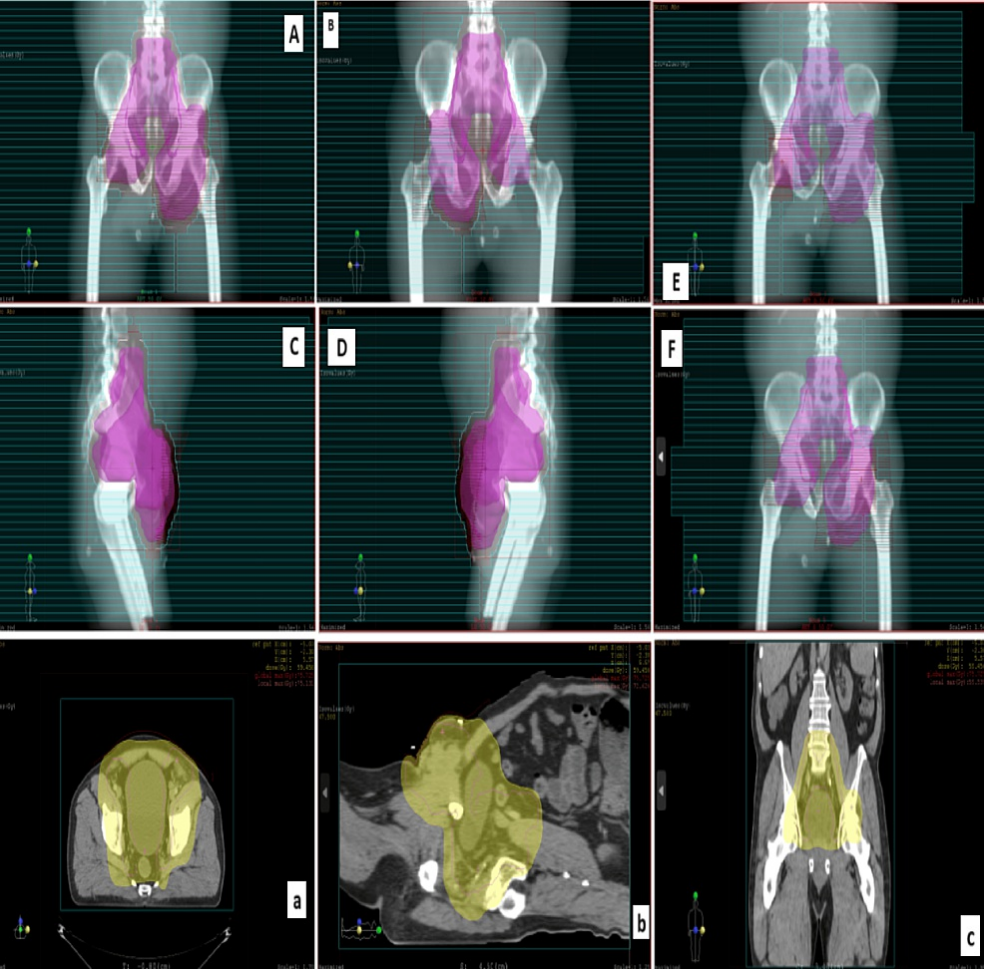
The treatment plan on the pelvis including the lower primary iliac, external iliac, internal iliac, obturator, and inguinal lymph node areas, receiving 50 Gy. (A) Anterior field; (B) posterior field; (C) right lateral field; (D) left lateral field; (E) right integrated boost; (F) left integrated boost and (a) axial section; (b) sagittal section; (c) frontal section

**Figure 5:**
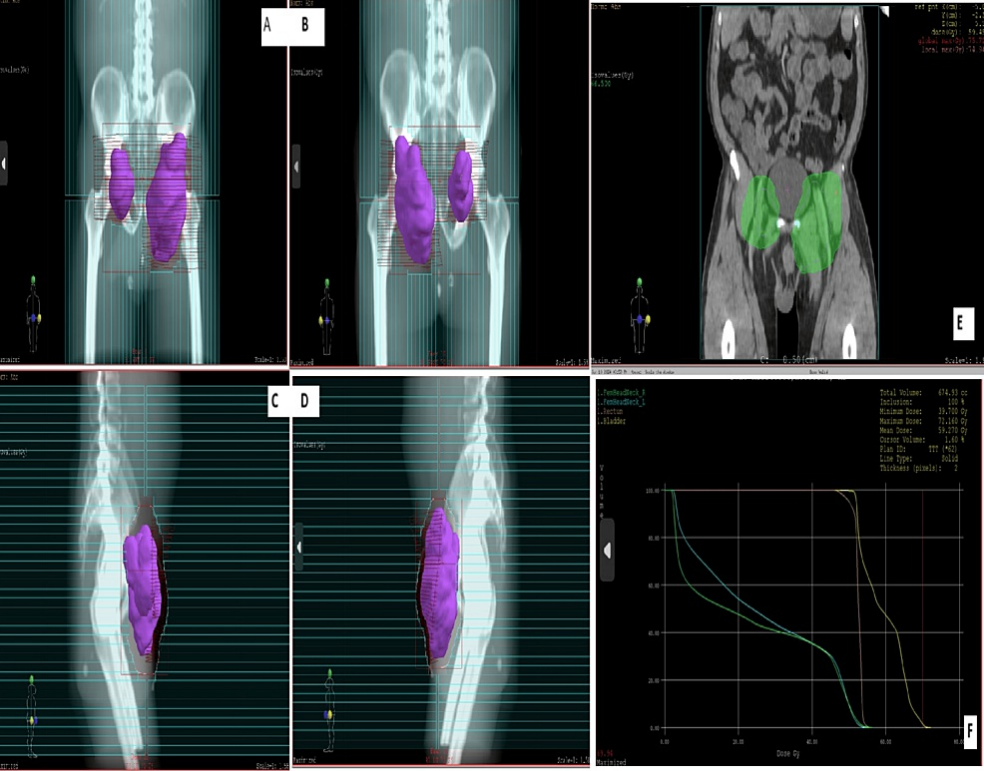
The treatment plan at the volume of pelvic lymphadenopathy receiving 70 Gy. (A) Anterior field; (B) posterior field; (C) right lateral field; (D) left lateral field; (E) frontal section; (F) dose volume histogram. The bolus is red in color.

**Figure 6:**
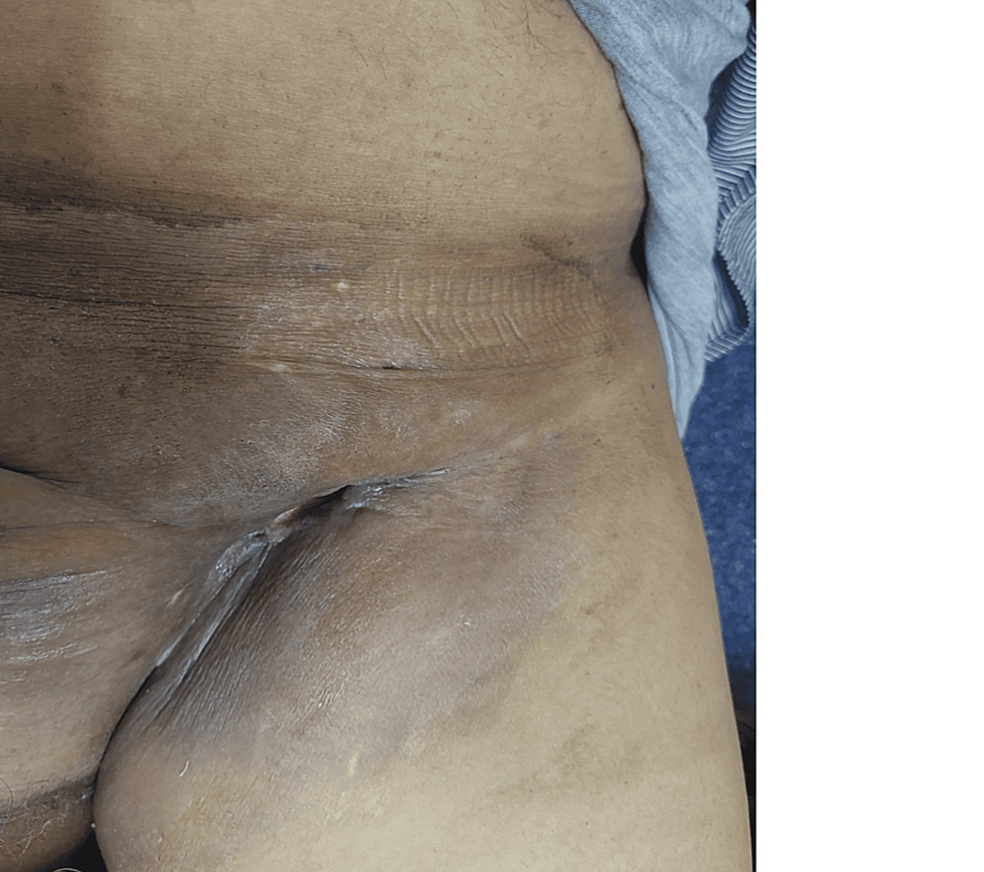
Almost complete disappearance of the ulcerated tumor in the left inguinal region.

The multidisciplinary meeting (MDM) decision was to offer concomitant chemo-radiotherapy, given that inguinal lymph node dissection (LND) was not feasible. The patient received radiotherapy as a total dose of 70 Gy in 35 fractions of 2 Gy by three-dimensional (3D) conformal radiation therapy with a photon at 6 and 18 MV energy (Figures 4-5), concomitantly with weekly platinum-based chemotherapy (cisplatin) with a good tolerance. After four months, there was almost complete disappearance of the ulcerated tumor in the left inguinal region in favor of a good evolution (Figure 6).

## Discussion

Cancer of the scrotum is a rare cancer; its incidence is approximately one per one million men per year. The most frequent histological type is SCC with almost 35% of all scrotal cancers. Other types exist including sarcoma, basal cell carcinoma, melanoma, and metastases [1,2].

SCC of the scrotum is principally due to poor hygiene, HPV, chronic skin irritation or inflammation, smoking, and ionizing or ultraviolet radiation. Some studies have shown that exposure to environmental carcinogens can cause this cancer, such as chimney soot, tars, kerosene, and certain petroleum products [3].

Scrotal SCC presents clinically as a localized papule or nodule often painless and sometimes ulcerated usually limited to one side of the scrotal region, with a predilection to the anteroinferior surface of the scrotum. It may occasionally spread to adjacent perineal skin, but rarely invades scrotal contents or the penis, and it progresses slowly, the reason why diagnosis is generally delayed. Nevertheless, some cases of rapid progression of scrotal SCC with ulceration and infection have been reported. In fact, inguinal lymph nodes are usually involved at the time of diagnosis, and can later spread to pelvic and para-aortic lymph nodes [4,5].

The imaging examination includes usually a pelvic CT scan and scrotal ultrasound. In recent days, magnetic resonance imaging (MRI) is the emerging investigation of choice in the diagnosis of scrotal diseases. A positron emission tomography (PET) scan should be considered if necessary such as in suspected pelvic lymph node involvement or beyond [6,7]. In our case, the stage of the disease was stage C with pelvic lymph node metastasis T2N+M0 and stage III as per Lowe’s staging of scrotal SCC.

The therapeutic management of scrotal SCC is controversial, and refers usually to the guidelines of penile SCC. Therefore, patients should always be managed in an MDM to ensure the best treatment is given to the patient [8,9].

The principal treatment of this cancer is surgical resection with clear margins, of at least 2-3 cm, in order to minimize the rate of disease recurrence. Sometimes for in situ SCC, other options can be used, such as the invasive carbon dioxide (CO_2_) laser and imiquimod, photodynamic therapy, and 5-fluorouracil. Sentinel lymph node biopsy or inguinal lymphadenectomy are recommended for patients suspected of having lymph node metastases. And in the case of clinically palpable lymph nodes or suspected of having metastatic lymph nodes on imaging, radical iliac lymph node dissection (ILND) should be performed [9,10].

In the case of very locally advanced tumors, neoadjuvant radiotherapy and chemotherapy are necessary to reduce tumor size and achieve complete resection. In our case, the scrotal lesion was excised with a negative margin. However, inguinal lymphadenectomy was not possible. Hence, concomitant adjuvant chemoradiotherapy was necessary. Data from the literature show improved survival in patients with scrotal SCC who have received radiotherapy combined with chemotherapy (methotrexate, bleomycin, cisplatin) for four cycles [10-12].

The prognosis of scrotal cancer depends on many factors such as patient age, tumor size, grade and stage, and the extent of surgery. Generally, patients with early-stage scrotal SCC have a good five-year survival (75%), while patients with more advanced stages have a poor prognosis. We also note that survival is poorer in patients with squamous histological features than in those with other histological subtypes. Johnson et al. analyzed 766 patients with scrotal cancer and found that median overall survival (OS) by histology was 180 months for sarcoma, 165 months for extramammary Paget's disease, 143 months for basal cell carcinoma, 136 months for melanoma, 115 months for SCC, and 114 months for adnexal skin tumor [12-14].

In our case, scrotal excision combined with inguinal definitive concomitant chemoradiotherapy achieved good loco-regional disease control at four months of follow-up.

## Conclusions

Scrotal SCC is a rare cancer. Awareness of risk factors and early access to medical care are essential for decreasing the prevalence of advanced diseases. Although there are no definitive guidelines for treating scrotal SCC, radical surgery remains the primary treatment approach, although concomitant chemoradiotherapy remains the ultimate curative treatment offering good outcomes for advanced stages.
